# Effects of Platelet-Rich Plasma on Proliferation, Viability, and Odontogenic Differentiation of Neural Crest Stem-Like Cells Derived from Human Dental Apical Papilla

**DOI:** 10.1155/2020/4671989

**Published:** 2020-05-09

**Authors:** Junyuan Li, Lusai Xiang, Chenyu Guan, Xin Yang, Xiaoli Hu, Xiaolei Zhang, Wen Zhang

**Affiliations:** ^1^The Medical Center of Stomatology, The First Affiliated Hospital of Jinan University, Guangzhou 510630, China; ^2^Guanghua School of Stomatology, Guangdong Provincial Key Laboratory of Stomatology, Stomatological Hospital, Sun Yat-sen University, Guangzhou 510055, China

## Abstract

**Objective:**

This study is aimed at evaluating the effects of platelet-rich plasma (PRP) on proliferation, viability, and odontogenic differentiation of neural crest stem-like cells (NCSCs) derived from human dental apical papilla.

**Materials and Methods:**

Cells from apical papillae were obtained and then induced to form neural spheres. The expression of NCSC markers p75NTR and HNK-1 in neural sphere cells was detected by immunofluorescence staining. Human PRP was prepared by a 2-step centrifugation method and activated by CaCl_2_ and thrombin. The concentrations of PDGF-BB and TGF-*β*1 in whole blood and PRP were measured by an ELISA kit. PRP in five different concentrations (0%, 2.5%, 5%, 10%, and 25%) was applied to culture NCSCs. On the 1^st^, 3^rd^, 5^th^, and 7^th^ days, cell proliferation was evaluated by CCK8. Cell viability was tested by a live/dead staining kit. mRNA and protein expression of DSPP and BMP4 were analyzed by RT-qPCR and western blot, respectively. Statistical analysis was performed by a one-way analysis of variance (ANOVA) test or *t*-test.

**Results:**

Dental apical papilla cells formed neural spheres, from which cells displayed positive expression of p75NTR and HNK-1. The concentrations of PDGF-BB and TGF-*β*1 in PRP were about 3.5-fold higher than those in whole blood. 5% and 10% PRP significantly promoted proliferation of NCSCs, while 25% and 50% PRP inhibited cell proliferation from Day 3 to Day 7. Low-concentration (2.5%, 5%, and 10%) PRP slightly improved viability of NCSCs on Day 7. On the other hand, high-concentration (25% and 50%) PRP significantly inhibited viability of NCSCs from Day 3 to Day 7. RT-qPCR and western blot results indicated that 10% PRP could promote odontogenic differentiation of NCSCs on Day 7. mRNA and protein expression of DSPP and BMP4 were significantly upregulated in the 10% PRP group compared to those in the control group (*P* < 0.05).

**Conclusions:**

PRP is a simply acquirable blood derivative which contains high concentration of growth factors like PDGF-BB and TGF-*β*1. PRP in a proper concentration could promote proliferation, viability, and odontogenic differentiation of NCSCs derived from human dental apical papilla.

## 1. Introduction

The American Association of Endodontics recommends using regenerative endodontic therapy (RET) in immature teeth with necrotic pulp [1, 2]. For dental pulp treatment, “RET,” “revascularization,” and “revitalization” are three synonyms, which are interchangeable [1]. The critical procedure of RET is to induce bleeding in periapical tissue. The blood clots can serve as scaffolds and release growth factors to guide stem cells from apical papilla (SCAP) into root canals for pulp regeneration [2–4]. It was reported that platelet-rich plasma (PRP) could be applied in RET to promote apical closure and root growth in immature permanent teeth [5–8].

PRP contains numerous growth factors, including epidermal growth factor (EGF), transforming growth factor beta 1 (TGF-*β*1), vascular endothelial growth factor (VEGF), platelet-derived growth factor-BB (PDGF-BB), and insulin-like growth factor (IGF) [9]. PRP is a favorable autologous scaffold that can facilitate cell proliferation and differentiation [10]. Dental pulp stem cells (DPSCs) cultured with a certain concentration of PRP exhibited preferable proliferation, migration, and mineralization [11, 12]. SCAP cultured with PRP expressed a high level of odontogenic markers such as dentin matrix protein 1 (DMP-1) and dentin sialophosphoprotein (DSPP) [13].

Both dental pulp and apical papilla develop from a cranial neural crest [14]. Some studies reported that NCSCs existed in dental pulp and apical papilla [15, 16]. When cultured under neural sphere-forming condition, dental pulp cells formed spheres in which cells expressed NCSC markers (p75NTR, HNK-1, Slug, Snail, Nestin, and Musashi1) [15]. These NCSCs in spheres had multiple differentiation capabilities, as they could differentiate into adipocytes, chondrocytes, osteoblasts, neurons, and smooth muscle cells. NCSCs obtained from apical papilla of immature teeth could differentiate into odontoblasts [16]. However, the influence of PRP on NCSCs is still unclear. Here, we studied effects of PRP on proliferation, viability, and odontogenic differentiation of NCSCs induced from human dental apical papilla.

## 2. Materials and Methods

### 2.1. Obtain NCSCs from Dental Apical Papilla

The experiment protocol of the current research was approved by the Ethical Review Committee of Sun Yat-sen University. Immature teeth were extracted from patients (aged 16-18 years) and then washed three times with saline to remove residual blood [17–19]. Dental papillae were separated from roots by a dental probe and placed in *α*-MEM (Gibco, Thermo Fisher Scientific, USA) with 10% fetal bovine serum (FBS), 100 U/ml penicillin, and 100 *μ*g/ml streptomycin. Subsequently, dental apical papillae were cut into small tissue pieces and then treated with collagenase type I and dispase type II for 30 minutes at 37°C. The tissue blocks were filtrated. After that, remaining liquid was centrifuged at 1000 rpm for 5 minutes. The supernatant was removed, and remaining cells were resuspended. The cells isolated from papillae were seeded on cell culture dishes and cultured in a condition of 5% CO_2_ at 37°C for two weeks. Next, these cells were cultured with neural sphere medium, i.e., DMEM/F12 was supplemented with N-2 (100x), B-27 (50x), 100 U/ml penicillin, 100 *μ*g/ml streptomycin, 20 ng/ml recombinant human EGF, and 20 ng/ml recombinant human FGF2 (Gibco, Thermo Fisher Scientific, USA). One week later, neural spheres were collected and detached into single cells by a StemPro Accutase Cell Dissociation Reagent (Life Technologies, Thermo Fisher Scientific) [20, 21]. These cells were seeded onto 1% Matrigel (Corning, Bedford, MA, USA) precoated dishes for subculture.

### 2.2. Immunofluorescence Assay

NCSCs at the 2^nd^ passage were digested, centrifuged, and placed on a 6-well culture plate at a density of 2 × 10^4^ per well. After 24 hours, 4% paraformaldehyde was used to fix the cells for 30 minutes, and 0.5% Triton X-100 was used to permeabilize the cells for 20 minutes. Later, the cells were blocked in 5% bovine serum albumin (BSA) for one hour and incubated with primary antibodies against p75NTR (8238S, Cell Signaling) and HNK-1 (ab187274, Abcam) overnight at 4°C. After 12 hours, the cells were probed with Goat Anti-Rabbit Alexa Fluor 488 (ab150077, Abcam) and Goat Anti-Mouse Alexa Fluor 647 (ab150115, Abcam) antibodies for one hour and then washed with phosphate buffer solution (PBS) for three times. Additionally, cell nuclei were counterstained with DAPI. Images were captured with an inverted fluorescence microscope.

### 2.3. Preparation of Human PRP

A total of four students from Sun Yat-sen University, China, donated their blood. The preparation of human PRP followed a standard protocol [22]. Briefly, whole blood was collected by a 100 ml syringe and centrifuged for 10 minutes at 180 g to separate plasma and blood cells. Next, plasma and blood cells were centrifuged again to three layers: the lower layer was blood cells. The upper layer was poor platelet plasma, and between the two layers was PRP. PRP was activated by adding CaCl_2_ (22 mM) and thrombin at 37°C for 40 minutes, and then, blood clots were removed. The exudate was centrifuged at 890 g for 10 minutes, and then, supernatant which contained growth factors was cryopreserved at −80°C for further application.

### 2.4. Growth Factor Concentration Measurement

The concentration of two growth factors (PDGF-BB and TGF-*β*1) in PRP and whole blood was tested by enzyme-linked immunosorbent assay (ELISA) kits (R&D Systems, Minneapolis, USA). Three repeats were carried out for each test; after that, the absorbance at 450 nm was detected and analyzed.

### 2.5. Cell Proliferation Assay

The 3^rd^ passage NCSCs were placed on 96-well cell culture plates with initial density of 2 × 10^3^ cells per well. Cell Counting Kit 8 (CCK8) (Dojindo, Tokyo, Japan) was applied to evaluate proliferation of those cells on the 1^st^, 3^rd^, 5^th^, and 7^th^ days after culture [17]. To evaluate the impacts of PRP on NCSC proliferation, five PRP concentrations (0%, 2.5%, 5%, 10%, and 25% PRP) were applied. The optical density (OD) was measured at 450 nm by a microplate reader (Thermo Scientific, UK).

### 2.6. Live/Dead Staining

The 3^rd^ passage NCSCs were laid upon 6-well plates precoated with 1% Matrigel at density of 5 × 10^4^ cells/well and cultured with neural sphere medium. 0%, 2.5%, 5%, 10%, 25%, and 50% PRP were added into the wells, respectively. On the 1^st^, 3^rd^, 5^th^, and 7^th^ days, a LIVE/DEAD Cell Imaging Kit (R37601, Invitrogen, Thermo Scientific, USA) was used to detect cell viability of NCSCs.

### 2.7. Quantitative Real-Time PCR (RT-qPCR)

Seven days after PRP treatment, total RNA was extracted by using a TRIzol reagent (Invitrogen, USA). The cDNA was synthesized by a RevertAid First Strand cDNA Synthesis Kit (Invitrogen, USA). RT-qPCR was completed by iQ SYBR Green Supermix (BioRad Laboratories, USA) with iQ5 thermal iCycler (BioRad Laboratories, USA). GAPDH was the control. The sequences of the target primers were the following: DSPP, forward 5′-TTTGGGCAGTAGCATGGGC-3′ and reverse 5′-CCATCTTGGGTATTCTCTTGCCT-3′; BMP4, forward 5′-ATGATTCCTGGTAACCGAATGC-3′ and reverse 5′-CCCCGTCTCAGGTATCAAACT-3′; and GAPDH, forward 5′-AAGGTGAAGGTCGGAGTCAA-3′ and reverse 5′-AATGAAGGGGTCATTGATGG-3′.

### 2.8. Western Blot

After culturing for seven days, cells in two groups were lysed in 0.1 ml RIPA buffer with PMSF (Invitrogen, Rockford, IL, USA) for 30 minutes and then centrifuged to collect supernatant. Target proteins were separated on 10% SDS-polyacrylamide gels (Beyotime Institute of Biotechnology, China) and transferred to PVDF membranes (Thermo Fisher Scientific, Carlsbad, CA, USA). PVDF membranes were incubated with primary antibodies at 4°C overnight. The primary antibodies were BMP4 (sc-12721, Santa Cruz Biotechnology Inc., Dallas, TX, USA) and DSPP (sc-73632). Later, PVDF membranes were incubated with secondary antibody (sc-516102) at 37°C for 2 hours. GAPDH (sc-47724) was set as the control.

### 2.9. Statistical Analysis

All data were presented as the mean ± standard deviation (SD). The one-way analysis of variance (ANOVA) test or *t*-test was performed. Statistical significance was defined as *P* < 0.05.

## 3. Results

### 3.1. Dental Apical Papilla Cells Formed Neural Spheres

**Figure 1:**
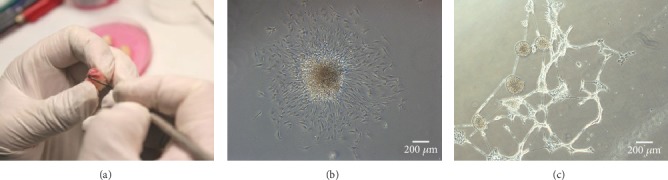
(a) Pink dental apical papillae were obtained. (b) Dental apical papilla cells formed colonies in one week. (c) Neural spheres were observed one week after induction.

The pink apical papillae were gently separated from teeth (Figure 1(a)). The dental apical papilla cells formed colonies one week after culture (Figure 1(b)). In sphere-forming medium containing FGF2, EGF, B-27, and N-2 supplements, the 3^rd^ passage dental papilla cells formed spheres within one week (Figure 1(c)).

### 3.2. Expression of NCSC Markers in Sphere-Forming Cells

**Figure 2:**
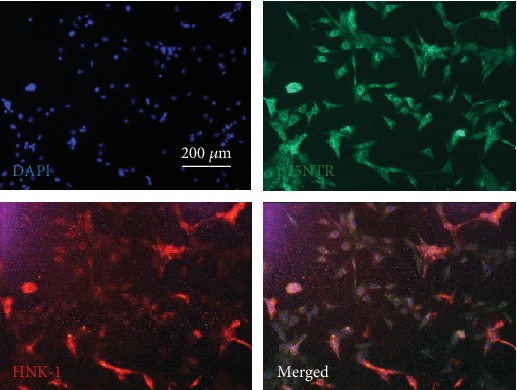
Neural sphere cells expressed p75NTR and HNK-1 in immunofluorescence assay.

Sphere-forming cells were visualized by immunofluorescence and verified positive expression of NCSC surface markers, including p75NTR and HNK-1 (Figure 2). These results indicated that the sphere-forming cells were NCSCs.

### 3.3. Character of Human PRP

Platelet counting in whole blood was 1.72 ± 0.13 × 10^8^/ml, while that in PRP was 7.02 ± 0.68 × 10^8^/ml. The number of platelets in PRP was almost 4.1-fold compared to that in whole blood. Mean red blood cell (RBC) counting reduced from 4.29 ± 0.85 × 10^6^/ml in whole blood to 0.13 ± 0.03 × 10^6^/ml in PRP (*P* < 0.01).

The activated PRP contained more PDGF-BB and TGF-*β*1 than whole blood (*P* < 0.01). The concentration of PDGF-BB in activated PRP (7.42 ± 0.44 ng/ml) was 3.4-fold higher than that in whole blood (2.20 ± 0.11 ng/ml). The concentration of TGF-*β*1 in PRP (41.38 ± 4.77 ng/ml) was 3.5-fold higher than that in whole blood (11.81 ± 1.70 ng/ml).

### 3.4. Influence of PRP on Proliferation of NCSCs

**Figure 3:**
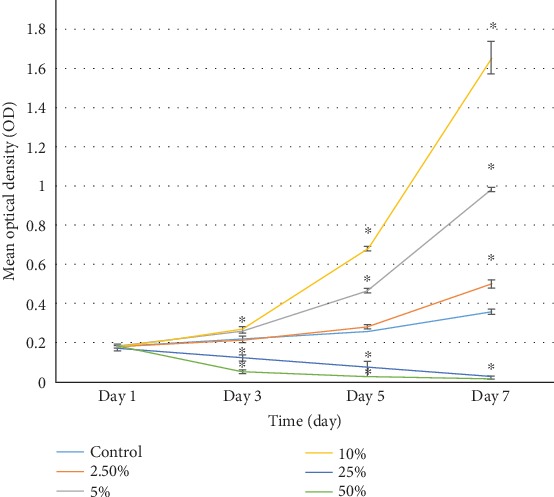
Influence of PRP concentration on NCSC proliferation.

As shown in Figure 3, average optical density (OD) indicated that the cell number was similar among five groups on Day 1. From Day 3 to Day 7, 5% and 10% PRP significantly promoted proliferation of NCSCs, while 25% and 50% PRP significantly inhibited cell proliferation. On Days 5 and 7, the OD value from high to low was 10%, 5%, 2.5%, 0%, 25%, and 50% PRP group, respectively.

### 3.5. Influence of PRP on Viability of NCSCs

**Figure 4:**
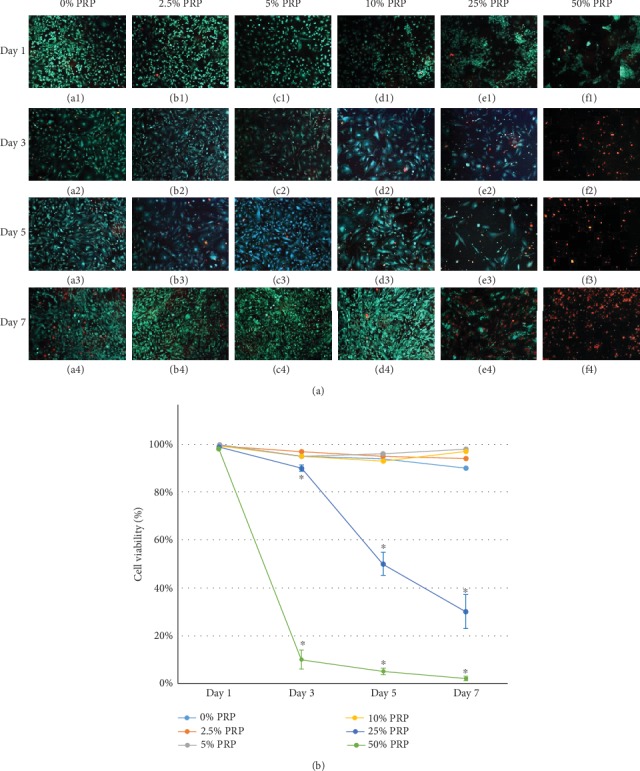
Influence of PRP on cell viability of NCSCs.

The fluorescent images of live/dead cells are shown in Figure 4(a), in which live cells were presented as green and dead cells as red. In Figure 4(b), 0%, 2.5%, 5%, and 10% PRP groups maintained significantly better cell viability than 25% and 50% PRP groups (*P* < 0.05) from Day 3 to Day 7. Low-concentration (2.5%-10%) PRP slightly improved viability of NCSCs compared with the control group on Day 7. Less than 10% NCSCs survived in the 50% PRP group from Day 3 to Day 7. Similarly, the viability decreased from 90% to 30% in the 25% PRP group over time. This result suggested that high-concentration (>25%) PRP decreased cell viability and was not recommended for NCSC culture.

### 3.6. 10% PRP Promoted Odontogenic Differentiation of NCSCs

**Figure 5:**
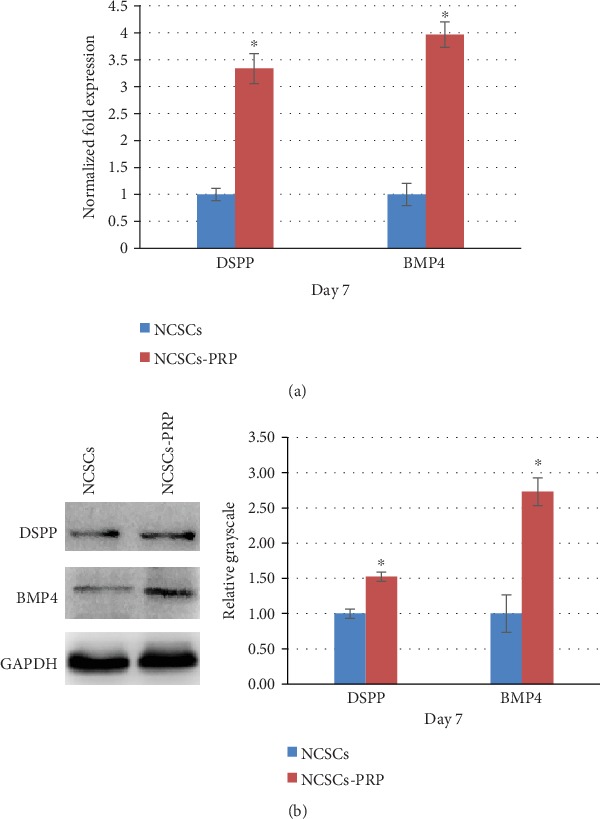
10% PRP promoted odontogenic differentiation of NCSCs. (a) In the NCSC-PRP group, the mRNA expression of DSPP and BMP4 increased 3.3-fold and 3.9-fold, respectively (*P* < 0.05). (b) In the NCSC-PRP group, the protein expression of DSPP and BMP4 increased 1.5-fold and 2.7-fold, respectively (*P* < 0.05).

10% PRP was selected to assess NCSC differentiation because it achieved optimal cell proliferation and viability as mentioned above. RT-qPCR was conducted to analyze key gene expression (DSPP and BMP4) involved in odontogenic differentiation of NCSCs (Figure 5(a)). Both DSPP and BMP4 mRNA expressions were significantly upregulated in the 10% PRP group compared with those in the control group (*P* < 0.05). Similarly, western blot results displayed that 10% PRP significantly promoted DSPP and BMP4 expression in the protein level (*P* < 0.05) (Figure 5(b)). Both RT-qPCR and western blot results suggested that 10% PRP promoted odontogenic differentiation of NCSCs *in vitro*.

## 4. Discussion

SCAP are progenitor cells of odontoblasts, which are crucial for dentin formation and root completion [23]. SCAP obtained from human third molars exhibited characteristics of neural crest-derived progenitor cells [16]. In the current study, the results displayed that SCAP formed neural spheres after directional induction using neural sphere-forming medium for one week. The similar phenomenon was observed in dental pulp cells using neural sphere-forming medium [15]. In our study, the neural sphere cells derived from apical papillae expressed p75NTR and HNK-1 which were classic markers of NCSCs [24, 25]. These results indicated that neural sphere cells derived from apical papillae were NCSCs.

Various studies reported that proliferation of dental cells could be influenced by different concentrations of PRP. A researcher pointed out that 1%-20% PRP significantly enhanced human DPSC proliferation in six days [26]. 2% to 10% PRP derived from human umbilical cord blood significantly stimulated proliferation of human DPSCs, while 2% PRP led to the highest proliferation in three days [27]. In another study, 0.5 and 1% PRP promoted proliferation of human DPSCs, but 5% PRP inhibited cell proliferation in five days [28]. Similar results were also observed in rat DPSCs; 1% and 10% PRP enhanced proliferation of DPSCs in ten days, but 50% PRP inhibited cell proliferation [12]. In the current experiment, 2.5%-10% PRP increased proliferation of NCSCs derived from the dental apical papilla, while 25% and 50% PRP inhibited cell proliferation in seven days. This result explained that low-concentration (2.5%-10%) PRP could promote NCSC proliferation, while high-concentration (25% and 50%) PRP gave an opposite result. The effects of PRP concentration on NCSCs are similar to DPSCs as mentioned above, but variance of PRP concentration might be attributed to the source of PRP and cell types.

There is only one direct evidence about the role of PRP on cell viability in the dental field. DPSCs exposed to 10% and 20% PRP acquired significantly (*P* < 0.05) higher viability than the cells cultured in 10% FBS on Day 4 [29]. Similar results were also observed in adipose tissue-derived mesenchymal stem cells, of which viability significantly increased when cultured with 5% and 20% PRP for 16 hours [30]. For Schwann cells, 2.5-10% PRP significantly promoted cell viability on Day 3 and Day 7 [31]. Besides, 5% PRP significantly increased viability of periodontal cells, while 50% PRP inhibited viability on Day 2 and Day 3 [32]. Creeper et al. pointed out that the high concentration of PRP could cause cytotoxic effect on periodontal ligament cells [33]. In our study, high-concentration (25% and 50%) PRP resulted in obvious low-level cell viability, while low-concentration (2.5%-10%) PRP slightly improved viability of NCSCs.

A few studies pointed out that PRP could improve odontogenic/osteogenic differentiation [13, 34, 35] and mineralization [11, 12] of dental stem cells. PRP promoted mRNA and protein expression of DSPP and DMP-1 in SCAP [13]. Because DSPP and DMP-1 are markers of odontoblasts, this result indicated that PRP could be a candidate for inducing SCAP into odontoblasts. Previous studies also reported that BMP4 acted as a crucial mesenchymal odontogenic signal during early tooth development [36]. Our results demonstrated that 10% PRP-treated NCSCs obtained higher expression of odontogenic differentiation markers, such as DSPP and BMP4. This result was consistent with the previous studies, which means NCSCs treated by certain concentration of PRP could obtain better odontogenic differentiation potential.

## 5. Conclusions

In this study, we have elucidated that PRP isolated from humans is a simply acquirable blood derivative which contains high concentration of growth factors like PDGF-BB and TGF-*β*1. Moreover, we found that PRP in proper concentration (2.5%-10%) could promote cell proliferation, viability, and odontogenic differentiation of NCSCs derived from human dental apical papilla. Therefore, these findings provide considerable evidence and valuable information to apply PRP in regenerative endodontic therapy.

## Data Availability

The data used to support the findings of this study are available from the corresponding author upon request.
